# Phenolic compound profiling and antioxidant potential of different types of *Schisandra henryi* in vitro cultures

**DOI:** 10.1007/s00253-024-13159-6

**Published:** 2024-05-07

**Authors:** Karolina Jafernik, Paweł Kubica, Marta Sharafan, Aleksandra Kruk, Magdalena Anna Malinowska, Sebastian Granica, Agnieszka Szopa

**Affiliations:** 1 Chair and Department of Pharmaceutical Botany, Medical College, Jagiellonian University, Medyczna 9 Str, 30-688 Cracow, Poland; 2 Department of Organic Chemistry and Technology, Faculty of Chemical Engineering and Technology, Cracow University of Technology, Warszawska 24 Str, 31-155 Cracow, Poland; 3 Microbiota Lab, Department of Pharmaceutical Biology, Medical University of Warsaw, Banacha 1 Str, 02-097 Warsaw, Poland

**Keywords:** *Schizandra*, Plant biotechnology, Phenolic acids, Procyanidins, Plant temporary immersion systems, Antioxidant potential

## Abstract

**Abstract:**

*Schisandra henryi* is an endemic species of medicinal potential known from traditional Chinese medicine. As part of this study, a complex biotechnological and phytochemical assessment was conducted on *S. henryi* with a focus on phenolic compounds and antioxidant profiling. The following in vitro cultures were tested: microshoot agar and callus, microshoot agitated, and suspension, along with the microshoot culture in PlantForm bioreactors. Qualitative profiling was performed by ultra-high-performance liquid chromatography with a photodiode array detector coupled with ion-trap mass spectrophotometry with electrospray ionization and then quantitative analysis by high-performance liquid chromatography with a diode array detector using standards. In the extracts, mainly the compounds from procyanidins were identified as well as phenolic acids (neochlorogenic acid, caffeic acid, protocatechuic acid) and catechin. The highest content of phenolic compounds was found for in vitro agar microshoot culture (max. total content 229.87 mg/100 g DW) and agitated culture (max. total content 22.82 mg/100 g DW). The max. TPC measured using the Folin-Ciocalteu assay was equal to 1240.51 mg GAE/100 g DW (agar microshoot culture). The extracts were evaluated for their antioxidant potential by the DPPH, FRAP, and chelate iron ion assays. The highest potential was indicated for agar microshoot culture (90% of inhibition and 59.31 nM/L TEAC, respectively). The research conducted on the polyphenol profiling and antioxidant potential of *S. henryi *in vitro culture extracts indicates the high therapeutic potential of this species.

**Key points:**

*• Different types of S. henryi in vitro cultures were compared for the first time. *

*• The S. henryi in vitro culture strong antioxidant potential was determined for the first time.*

*• The polyphenol profiling of different types of S. henryi in vitro cultures was shown.*

**Supplementary Information:**

The online version contains supplementary material available at 10.1007/s00253-024-13159-6.

## Introduction

Polyphenols are one of the most scientifically desirable and useful groups of compounds obtained from plants. They show a number of medicinal properties including, antioxidant, anti-inflammatory, and antimicrobial (Waterhouse 2002; Prior et al. 2005; Grace 2007). This is evident as they are widely used in medicinal and cosmetic preparations (Srinivasan et al. 2002; de Lima Cherubim et al. 2020). A number of studies conducted on these chemical compounds confirm their effective usage in anticancer therapies and treatments for dysfunctions related to the musculoskeletal system, as well as in neurodegenerative disorders (Gomes et al. 2003; Grace 2007; Roleira et al. 2015; Rosa et al. 2016). Polyphenols are the most common group of plant secondary metabolites. These compounds include phenolic acids, flavonoids, proanthocyanidins, catechins, and tannins (Grace 2007). The plant biotechnology studies present that plant in vitro cultures can be an efficient source of polyphenol substances. The possibilities created by different plant biotechnology methods create effective high production methods of phenolic compounds in the tissue cultures of various plant species. Thanks to the optimization of processes related to the culture type selection, cultivation conditions, and breeding cycle duration, they can be proposed as an alternative to obtaining unpolluted, ecological source of polyphenols. Biomass of in vitro cultures is now increasingly used in cosmetic, nutraceutical, and pharmaceutical formulations as a source of antioxidants, which is related to the production of polyphenolic compounds (Barbulova et al. 2014; Elansary et al. 2019; Szopa et al. 2019c, 2020; Jafernik et al. 2020; García-Pérez et al. 2020; Hasnain et al. 2022; Zheleznichenko et al. 2023).

The main representative species of the Schisandraceae family is *Schisandra chinensis* Turcz. Baill. It is a species widely used in medicine due to its therapeutic properties (Szopa et al. 2012; Kam Ming et al. 2015). Its healing potential has been known in traditional Chinese medicine (TCM), and nowadays, the *Schisandra* fruit has been introduced to different pharmacopeias around the world (World Health Organization 2007; Li-jia et al. 2012; Kam Ming et al. 2015; European Directorate for the Quality of Medicines. 2017; Szopa et al. 2019a). *S. chinensis* fruits show primarily hepatoprotective effect, but they have also been tested for anticancer or anti-inflammatory properties (Choi et al. 2006; Huyke et al. 2007; Xue et al. 2010; Li-jia et al. 2012). The major groups of substances found in the *Schisandra* family are lignans, primarily dibenzocyclooctadiene lignans (Ko et al. 1995; Ip et al. 1996; Smejkal et al. 2010). Recently, increasingly more studies show that polyphenols in the *Schisandra* plant extracts also play a significant role contributing their biological power (Whiting 1990; Chiu et al. 2002; Choi et al. 2006; Havel et al. 2008).

*S. henryi* C.B. Clarke is an endemic plant of *Schisandra* genus. It occurs naturally in Yunnan Province in China. *S. henryi* is used in TCM in Asia, but it is practically unknown in European and American countries (Saunders 2000). The small number of studies on the *S. henryi* species has been focused on the phytochemical profiling (Liu et al. 1984; Sen et al. 1984; Jia-Sen et al. 1988; Iu et al. 2009; Chen et al. 2010; Bin et al. 2020). Some studies on biological properties have proved the neuroprotective, cytotoxic, and antiproliferative properties. It has been presented that triterpenoids isolated from *S. henryi* seeds can inhibit the growth of the P-388 cell line (Jia-Sen et al. 1988). Another study identified that dibenzocyclooctadiene lignans from dried stems may have the ability to stop the increase of leukemia and HeLA cell lines (Chen et al. 2003). Another study focused on the isolation of 12 schinotriterpenoids from the stems and leaves, which were proved to have the neuroprotective properties in tests of apoptosis induction by corticosterone in PC12 cells. The *S. henryi* extracts have not been tested for antioxidant potential. Furthermore, there is only one scientific study on the in vitro cultures of *S. henryi* which were proven to produce lignans (Jafernik et al. 2020).

The aim of this study was to investigate the polyphenol profile and to assess *S. henryi*’s antioxidant power. The biomass of in vitro cultures of *S. henryi* for this aspects was analyzed for the first time. A qualitative and quantitative analysis was conducted on the phytochemical profile of polyphenols found in the biomass; several types of in vitro cultures were assessed including agar microshoot and callus, microshoot in bioreactor PlantForm, agitated microshoot, and suspension cultures.

Ultra-high-performance liquid chromatography with a photodiode array detector coupled with ion-trap mass spectrophotometry with electrospray ionization (UHPLC-DAD-ESI-MS3) and high-performance liquid chromatography with a diode array detector (HPLC–DAD) were used for the analyses. In addition, the total content of polyphenols was measured using the Folin-Ciocalteu method. The antioxidant potential was assessed using 2,2-diphenyl-1-picrylhydrazine hydrate (DPPH), ferric reducing antioxidant power (FRAP), and ferrous ion chelating (FIC) assays.

## Materials and methods

### Initiation of in vitro culture

The plant material for initiation in vitro culture *S. henryi* was obtained from Clematis arboretum (CLEMATIS Sp. z o.o., Pruszków, Poland). The leaf buds which were used to initiate in vitro cultures were harvested in April 2018.

The *S. henryi* leaf buds were greased with 70% ethanol (30 s) and then assessed to further sterilization. HgCl_2_ (mercuric chloride II) at a concentration of 0.1% for 6 min was used for sterilization. Sterile buds were flushed with sterile redefined water and transferred to agar media according to Murashige and Skoog (MS)(Murashige and Skoog 1962) enhanced with plant growth regulators (PGRs): cytokinin 1 mg/L BA (6-benzyladenine, Sigma-Aldrich) and auxin 0.5 mg/L NAA (1-naphthaleneacetic acid, Sigma-Aldrich) (Bhojwani and Razdan 1996; Mohan 2016). Microshoots appeared after about 4 weeks, followed by stable in vitro cultures after about 8 weeks. Callus tissue appeared accompanying the initial shoots. The callus tissue was removed and produced on the MS medium containing 1 mg/L BA and 1 mg/L NAA. Cultures were propagated by transfer to fresh medium every 30 days.

### Experimental undifferentiated cultures

#### Callus culture

**Fig. 1:**
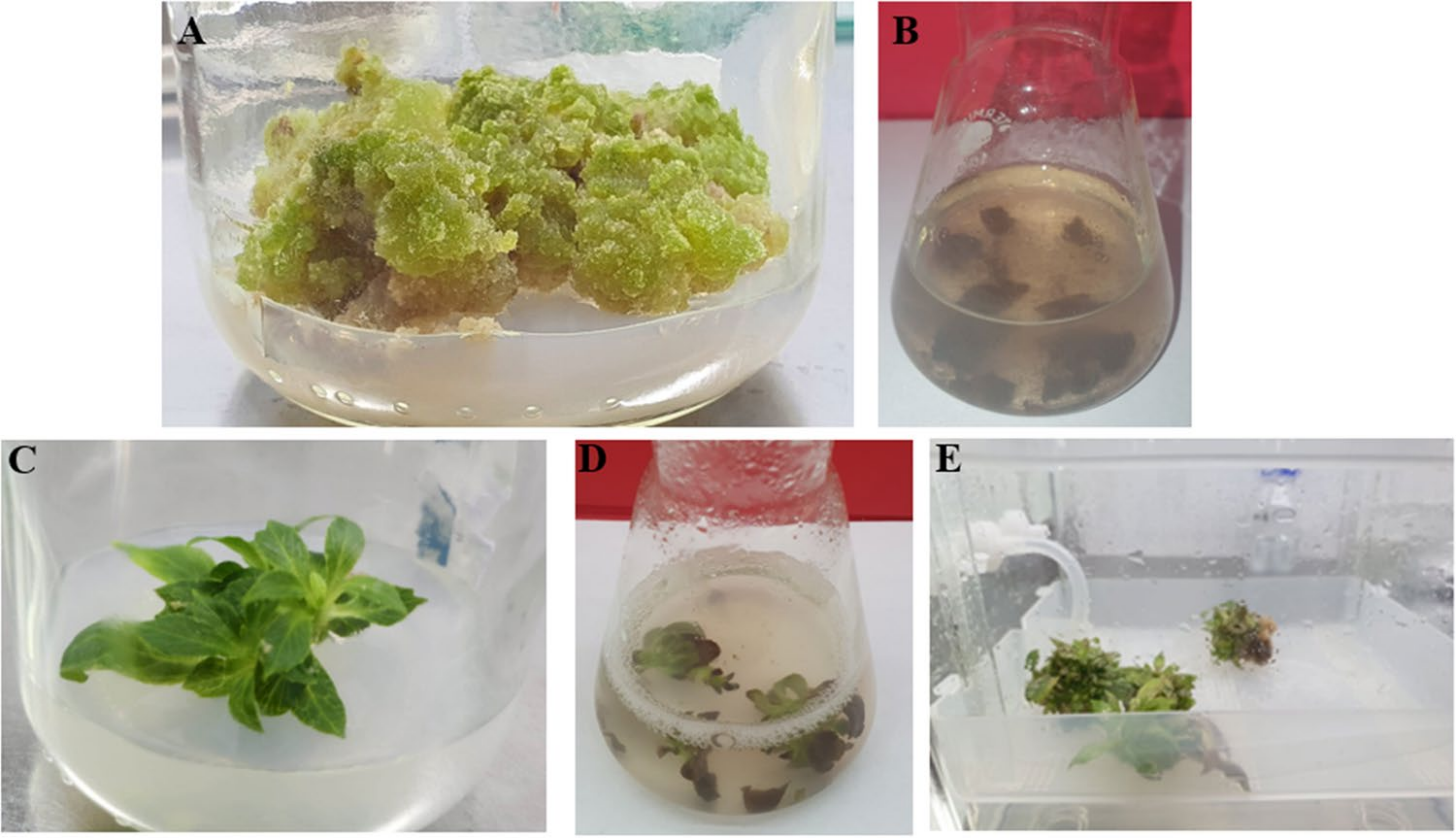
Morphological appearance of tested types in vitro cultures of *S. henryi*: **A** callus culture; **B** suspension culture; **C** agar microshoot culture; **D** microshoot agitated culture; **E** microshoot culture in PlantForm bioreactor

The study involved the cultivation of callus (Fig. 1A) on MS agar medium with the inclusion of 0.5 mg/L BA and 2 mg/L IBA (indole-3-butyric acid, Sigma-Aldrich), 3% (*w/v*) sucrose, and 0.72% (*w/v*) agar (plant agar, Duchefa, Haarlem, the Netherlands). The cultures were maintained at 25 ± 2 °C, under continuous artificial illumination with white LED light, with a photosynthetic photon flux density (PPFD) of 40 μmol m^−2^ s^−1^ and cultivated over the 30-day breeding cycles. The agar callus culture was carried out in glass containers intended for plant tissues (No. V8630, Sigma-Aldrich, Algés, Portugal). The 0.5 g of inoculum per one container was used for the experiment (three series, *n* = 10).

#### Suspension culture

The inoculum for suspension culture (Fig. 1B) consisted of 1.5 g of callus per Erlenmeyer flask (*v* = 100 mL) containing 50 mL of MS liquid medium with 0.5 mg/L BA, 2 mg/L IBA, and 3% sucrose (*w/v*). The cultures were agitated on the shaker at 120 rpm with a vibration amplitude of 35 mm, (Ohaus, model SHEX1619DG, Nänikon, Switzerland) under the same conditions as supposed above (three series, *n* = 10).

### Experimental microshoot cultures

#### Microshoot agar culture

The microshoot culture (Fig. 1C) was maintained on the MS medium inclusive 0.5 mg/L BA and 2 mg/L IBA, 3% (*w/v*) sucrose, and 0.72% (*w/v*) agar, at 25 ± 2 °C, under continuous artificial illumination with white LED light (PPFD of 40 μmol m^−2^ s^−1^). Agar microshoot cultures were grown in glass containers intended for plant tissues (No. V8630c, Duchefa Biochemie, Haarlem, the Netherlands). The 0.5 g of fresh microshoots (inoculum) per one container was used. The growth period lasted 30 days (three series, *n* = 10).

#### Microshoot agitated culture

Agitated microshoot culture was maintained on MS medium which 0.5 mg/L BA, 2 mg/L IBA, and 3% sucrose (*w/v*) (Fig. 1D). For cultivation, the Erlenmeyer flasks (*V* = 100 mL) were used. The inoculum consisted of 0.5 g of fresh microshoots per flask containing 50 mL of liquid medium. The cultures were agitated on the shaker at 120 rpm with a vibration amplitude of 35 mm, (Ohaus, model SHEX1619DG, Nänikon, Switzerland) over 30-day growth periods under the same conditions as described above (three series, *n* = 10).

#### Microshoot culture in bioreactor

The microshoot cultures were tested for cultivation in the PlantForm temporary immersion systems (PlantForm, Hjärup, Sweden) (Fig. 1E). The applied inoculum was 5 g of microshoots per one container with 500 mL of MS liquid medium with 0.5 mg/L BA and 2 mg/L IBA and 3% sucrose (*w/v*). The immersion cycle was set to 5 min every 1.5 h, with an aeration rate of 1.0 vvm. The cultures were maintained at the same physical conditions as described above over 30-day growth periods (three series, *n* = 3).

### Extraction

The biomass of in vitro tissues was lyophilized (Labconco Corporation, Kansas City, MO, USA). For one sample, the 0.3 g DW (dry weight) of plant tissue was extracted by sonication in ultrasonic bath (Sonic-2, POLSONIC, Warsaw, Poland; ultrasound power 2 × 100 W, 40 kHz, volume 1.6 L) twice for 30 min at 25 ± 2 °C. The extracts were centrifuged (MPW-223E, MPW, Warsaw, Poland) at 4000 rpm for 5 min. The extracts were then filtered through syringe filters (0.22 Pm Millex® GP, Millipore, Merck, Darmstadt, Germany) (each sample in three repetitions).

### UHPLC-DAD-ESI-MS^3^ analyses

The UHPLC-DAD-MS^3^ analysis was achieved on an UHPLC-3000 RS system (Dionex, Leipzig, Germany) equipped with a DAD detector and splitless connection with an AmaZon SL ion trap mass spectrometer with an ESI interface (Bruker Daltonik GmbH, Bremen, Germany). The UV spectra were obtained over the range of 200–450 nm. The parameters of the MS unit were as follows: nebulizer pressure 40 psi, drying gas flow rate 9 L/min, nitrogen gas temperature 134 °C, and capillary voltage 4.5 kV. The mass spectra were registered by scanning from *m*/*z* 70 to 2200. Kinetex XB-C18 chromatography columns were used (Phenomenex, Torrance, California, USA, 150 mm × 3.0 mm × 2.6 µm for phytochemical analysis of control sample; 150 mm × 2.1 mm × 1.7 µm for batch culture extract analysis). The mobile phase (A) was H_2_O/formic acid (100:0.1, *v/v*), and the mobile phase (B) was acetonitrile/formic acid (100:0.1, *v/v*). All solvents were analytical or LC–MS grade. The gradient program and the flow rate were 0–20 min 1–26% B, 20–80 min 26–75% B, 80–90 min 75–100% B, and 0.3 mL/min, respectively. The column oven temperature was set to 25 °C. The injection volume was 3 µL.

### HPLC–DAD analyses

Following UHPLC-DAD-ESI-MS^3^, the HPLC–DAD analysis was performed to quantify the detected compounds. The analysis was performed using the validated method (Ellnain-Wojtaszek and Zgorka 1999) of applying Merck-Hitachi liquid chromatograph (LaChrom Elite, Darmstadt, Germany) with a DAD L-2455 detector. Separation was performed on a Purospher RP-18 column (250 × 4 mm; 5 μm, Merck, Germany). The mobile phase consisted of A-methanol, 0.5% acetic acid 1:4, and B-methanol (*v/v*). The flow rate was 1 mL/min at 25 °C. The gradient was 100% A for 0–20 min, 100–80% A for 20–35 min, 80–70% A and 20–30% B for 35–45 min, 70–60% A and 30–40% B for 45–55 min, 60–50% A and 40–50% B for 55–60 min, 50–25% A and 50–75% B for 60–65 min, 25–0% A and 75–100% B for 65–70 min, 0–0% A and 100–100% B for 70–75 min, 0–100% A and 100–0% B for 75–80 min, and 100–100% A and 0–0% B for 80–90 min. The injection volume was 10 μL. The UV spectra were captured over the range of 200–400 nm; the compounds were identified at 254 nm. Identification and quantification were accomplished by comparing the retention times, UV spectra, and the verified fragmentation spectra by UHPLC-DAD-ESI-MS^3^. Quantification was performed on the basis of calibration curve method. The results are expressed in mg/100 g DW (dry weight). Qualitatively determined compounds being derivatives of protocatechuic and caffeic acids were quantitatively converted to protocatechuic and caffeic acids.

### Total polyphenol assay (TPC)

The TPC was determined using the method outlined by Kubica et al. (2022). Each sample solution was prepared by mixing 0.2 mL of Folin-Ciocalteu reagent, 100 µL of the sample with, 2 mL of H_2_O, and 1 mL of 15% Na_2_CO_3_. After a 2-h incubation at room temperature, the absorbance of the samples was measured using a Nanocolor UV/VIS spectrophotometer (Macherey–Nagel, Düren, Germany) at 765 nm. The total phenolic content was expressed as gallic acid equivalent (GAE) in 1 g of extract ± standard deviation (SD). The calibration curve, established with six different concentrations of gallic acid (0.0625, 0.125, 0.25, 0.5, 1.0, and 2.0 (mg/mL)), was utilized. The measurements of the extracts total polyphenolic content were conducted in triplicate.

### The antioxidant potential

#### DPPH (1,1-diphenyl-2-picryl-hydrazyl) assay

The DPPH method, as previously described by Kubica et al. (2022), was employed to evaluate the total antioxidant potential of the tested extracts. In this process, 0.5 mL of each extract, spanning concentrations from 0.0625 to 2 mg/mL, was mixed with 3 mL of freshly prepared methanol–DPPH solution (0.1 mM). Following a 20-min mixing period, the absorbance of the solutions was measured at 517 nm by Nanocolor UV/VIS spectrophotometer (Macherey–Nagel). Butylated hydroxytoluene (BHT), matched in concentrations to the extract, was used as a reference. The results, representing the mean radical scavenging activity (%) ± SD, were obtained from three independent trials.

#### FRAP (ferric reducing antioxidant power) assay

The ferric reducing antioxidant power (FRAP) of CuO-NPs was evaluated using the procedure outlined by Iqbal et al. (2022). Initially, 10 μL of the test sample was mixed with 190 μL of a FRAP solution containing 2,4,6-tri(2-pyridyl)-s-triazine (TPTZ; 10 mM), ferric chloride hexahydrate (FeCl_3_·6H_2_O; 20 mM), and acetate buffer (300 mM) with a pH of 3.6, in a 10:1:1 (*v/v*/v) ratio. The reaction mixture was allowed to incubate at room temperature for 15 min, followed by measuring the absorbance at 630 nm using a Nanocolor UV/VIS spectrophotometer (Macherey Nagel). This activity was quantified as TEAC (Trolox equivalent antioxidant capacity). Trolox standards were employed at concentrations of 10, 20, 30, 40, 50, and 60 μmol/L to generate the standard curve equation *y* = 0.0093*x* + 0.0094 (*R*^2^ = 0.9936). The assay was carried out in triplicate.

#### FIC (ferrous ion chelating) assay

The Fe^2+^ chelating activity of the examined extracts was determined by measuring the formation of the Fe^2+^–ferrozine complex, as outlined in a previously described method (Kubica et al. 2022). Each sample, ranging in concentrations from 0.0625 to 2 mg/mL was mixed with 1 mL of extract, 0.5 mL of methanol, and 0.05 mL of 2 mM FeCl_2_. The initiation of complex formation occurred with the addition of 0.1 mL of 5 mM ferrozine. After thorough shaking, the mixtures were allowed to stand undisturbed for 10 min at room temperature. Following this, the absorbance values of the samples were measured at 562 nm using a Nanocolor UV/VIS spectrophotometer (Macherey–Nagel). A reference solution of ethylenediaminetetraacetic acid (EDTA, 2 mg/mL) was employed. The experiments were carried out in triplicate, and the results were presented as average values, reported as the mean inhibition of the Fe^2+^–ferrozine complex formation (%) ± SD.

### Statistical analysis

The experiments were performed in triplicate and the data was evaluated to find out mean values and standard deviation (means ± SD).

## Results

### Polyphenol phytochemical profiling

Qualitative analysis of polyphenols found in methanolic extracts from biomass of experimental in vitro cultures verified the presence of different phenolic compounds depending on the type of tested culture (Table 1, Supplemental Fig. S1). The 12 compounds were identified in the extracts of callus cultures, and two compounds in suspension cultures (Table 1). In the microshoot cultures, the 13 compounds were identified for agar culture, 24 compounds for agitated culture, and seven compounds in cultures grown in PlantForm bioreactors (Table 1, Supplemental Fig. S1). The compounds were classified into different phytochemical groups: phenolic acids and their derivatives, along with procyanidins and their derivatives (Table 1). Most compounds were identified or tentatively identified based on a comparison with the literature reports or available chemical standards. The parameters considered were UV–VIS spectra, MS, and MS^2^ profiles obtained for major *m*/*z* signals recorded in negative ion mode. In the extracts unknown compounds were also indicated (Supplemental Fig. S1).

**Table 1:** Phenolic profile of biomass extracts of *S. henryi* leaves and different types of in vitro cultures determined by the UHPLC-DAD-ESI-MS^3^

Compound	Retention time (min)	UV–Vis	Parent ion (*m*/*z*)	Reference ion	Daughter MS^2^ ions
Protocatechuic acid *O*-hexoside^1,3,4^*	8.7	< 210	315	[M-H]^−^	153b, 195, 225, 279
Procyanidin trimer type C isomer^1,3,4^	9.2	< 210, 283	865	[M-H]^−^	286, 289, 363, 407, 451, 543, 575, 577, 695b
Neochlorogenic acid^3,4^	10.7	< 210, 293sh, 324	353	[M-H]^−^	179, 191b
Procyanidin dimer type A isomer^4^	10.7	< 210, 323	575	[M-H]^−^	287, 289, 307, 407b, 423, 499
Procyanidin trimer type C isomer^3,4^	11.8	< 210, 278	865	[M-H]^−^	287, 289, 363, 407, 451, 543, 695b
Coumaroylquinic acid isomer^1,3,4^	12.4	< 210, 279, 310	337	[M-H]^−^	163b
Procyanidin dimer type B isomer^1,3,4^	12.4	< 210, 279, 310	577	[M-H]^−^	245, 289, 407, 425, 451, 559
Procyanidin dimer type B isomer^1,2,3,4,5^	12.7	< 210, 279	577	[M-H]^−^	245, 289, 407b, 421, 451, 559
Procyanidin tetramer isomer^4^	12.7	< 210, 279	1153	[M-H]^−^	407, 425, 578b, 683
Procyanidin dimer type B isomer^5^	12.9	< 210, 282	577	[M-H]^−^	245, 289, 407, 425b, 451, 499
Catechin^1,2,3,4,5^	13.2	< 210, 279	289	[M-H]^−^	205, 245b
Caffeic acid derivative^3^	13.9	< 210, 279	431	[M-H]^−^	179b
Procyanidin trimer type C isomer^1,3,4^	13.9	< 210, 279	865	[M-H]^−^	245, 363, 407, 425, 451, 543, 577b, 695,677
Procyanidin dimer type B isomer^1^	13.9	< 210, 279	577	[M-H]^−^	289, 407b, 425, 451, 559
Procyanidin tetramer isomer^1,3,4^	14.3	< 210, 268	1153	[M-H]^−^	407, 577b, 695, 769, 865, 984
Procyanidin tetramer isomer^4,5^	14.9	< 210, 279	1153	[M-H]^−^	289, 344, 407, 413, 452, 575, 695, 739, 866, 984b
Procyanidin tetramer isomer^1,3^	15	< 210, 279	1153	[M-H]^−^	407, 451, 534, 576, 661, 865, 984
Procyanidin dimer type B isomer^4^	15.3	< 210, 279, 310	591	[M-H]^−^	245, 289, 301, 407, 439, 465, 573
Coumaroylquinic acid isomer^1,4,5^	15.3	< 210, 280, 310	337	[M-H]^−^	163, 191b
Procyanidin dimer type B isomer^1,3,4,5^	15.6	< 210, 278	577	[M-H]^−^	289, 407b, 425, 451, 559
Procyanidin trimer type C isomer^1,3,4,5^	15.6	< 210, 278	865	[M-H]^−^	245, 289, 407, 425, 451, 575, 695b
Procyanidin pentamer isomer^4^	16	< 210, 280	720	[M-2H]^2^	245, 289, 407, 415, 425, 451, 489, 550, 577
Procyanidin tetramer isomer^1,3^	16.7	< 210, 279	1153	[M-H]^−^	407, 451, 534, 576, 661, 865, 984
Procyanidin dimer type B isomer^1,4^	17.1	< 210, 278	577	[M-H]^**−**^	289. 407, 425b, 451, 539
Procyanidin trimer type C isomer^1,4^	17.1	< 210, 279	865	[M-H]^**−**^	245, 289, 363, 407, 425, 451, 543, 577, 695b
Procyanidin dimer type B isomer^4,5^	17.2	214, 276	613	[M + HCOOH-H]^**−**^	577b
Procyanidin tetramer isomer^4,5^	17.7	< 210, 279	1153	[M-H]^**−**^	289, 344, 407, 413, 452, 575, 695, 739, 866, 984b
Procyanidin tetramer isomer^4^	18.2	< 210, 279	1153	[M-H]^**−**^	289, 344, 407, 413, 452, 575, 695, 739, 866, 984b
Procyanidin pentamer isomer^4^	18.7	215, 272	720	[M-2H]^2^	245, 289, 407, 415, 425, 451, 489, 550, 577

*The presence of compounds in biomass extracts in cultures was confirmed: ^1^callus, ^2^suspension, ^3^agar microshoots, ^4^agitated microshoots, microshoots in bioreactors (see Supplemental Figure S1)

Among the detected compounds, the dominant group was phenolic acids. Compound no. 1, present in agar callus, agar, and agitated microshoots, with the main ion at *m*/*z* 315, was characterized by fragmentation leading to the loss of hexose (162 Da) to form a protocatechuic acid ion (at *m*/*z* 153) (Wang et al. 2017). Compound no. 3 (present in agar and agitated microshoots) with the main ion at *m*/*z* 353 based on fragmentation to caffeic acid ion (*m*/*z* 179) and quinic acid ion (*m*/*z* 191) and retention time was recognized as neochlorogenic acid (Szopa et al. 2020); signals detected in extracts form  callus and agar and agitated microshoots, with the main ion at *m*/*z* 337 and characteristic fragmentation leading to quinic acid ion (*m*/*z* 191) were classified as coumaroylquinic acid isomers (Szopa et al. 2020). In agar microshoots, caffeic acid derivative (*m*/*z* 431) with fragmentation to ion of caffeic acid (*m*/*z* 179) was also observed (Szopa et al. 2020). Phenolic acid derivatives showed typical UV–VIS maxima in 279–293 and 310–324 nm.

The subsequent compound group was procyanidins. Compounds with the main ion at *m*/*z* 577 were subjected to MS^2^ fragmentation, leading to the ion pattern characteristic for dimeric B-type proanthocyanidins (Fecka et al. 2015). Compound no. 4 in agitated microshoots also had dimeric structure, due to main (ion *m*/*z* 575) and fragmentation pattern, which was classified as A-type procyanidin. An analogous MS^2^ fragmentation pattern with larger ions, including dimeric procyanidin (*m*/*z* 577) ion, was observed for signals with the main ion at *m*/*z* 865. Based on the available literature, these compounds were identified as trimeric B-type proanthocyanidins (Fecka et al. 2015). In spectra were observed signals at *m*/*z* 1153 and 720 (reference ion [M-2H]^2−^), which have been classified based on MS^2^ fragmentation pattern as procyanidin tetramers and pentamers, respectively (Fecka et al. 2015). In samples, catechin (*m*/*z* 289) was also observed as a procyanidin precursor (compared with analytical standard). The common feature of all procyanidins was their fragmentation, resulting in the production of catechin ion at *m*/*z* 289 and U*V*–*V*IS maxima 268–310 nm.

The quantification of dominant compounds performed with DAD-HPLC method for in vitro culture extracts was done for neochlorogenic acid, caffeic acid, and protocatechuic acid, as well as catechin (Table 2).

**Table 2:** Results of quantitative analysis (mg/100 g DW ± SD) of phenolic compounds in the tested types of *S. henryi* in vitro cultures

Phenolic compounds	Type of culture				
	Undifferentiated culture		Microshoot culture		
	Callus	Suspension	Agar microshoot	Agitated microshoot	Microshoots in bioreactor
Neochlorogenic acid	nd*	nd	210.13 ± 12.45	22.82 ± 1.57	nd
Caffeic acid	nd	nd	3.20 ± 0.45	nd	nd
Protocatechuic acid	7.51 ± 2.01	0.07 ± 0.02	16.54 ± 1.29	18.11 ± 2.39	nd
Catechin	22.51 ± 2.57	65.16 ± 3.98	112.56 ± 3.52	65.79 ± 4.36	124.25 ± 2.35

*nd not detected

In agar callus culture, the content of protocatechuic acid was 7.51 mg/100 g DW and catechin 22.51 mg/100 g DW (Table 2). For the tested extracts from suspension cultures, the content of protocatechuic acid was 0.07 mg/100 g DW and catechin 65.16 mg/100 g DW (Table 2). In the microshoot agar cultures, neochlorogenic acid (210.13 mg/100 g DW), caffeic acid (3.20 mg/100 g DW), protocatechuic acid (16.54 mg/100 g DW), and catechin (112.56 mg/100 g DW) were quantified. In the  extracts from suspension culture, the following phenolic compounds were detected: protocatechuic acid (0.07 mg/100 g DW) and catechin (65.16 mg/100 g DW) (Table 2). The content of these compound in the agitated cultures was 22.82 mg/100 g DW (neochlorogenic acid), 18.11 mg/100 g DW (protocatechuic acid), 65.79 mg/100 g DW (catechin); protocatechuic acid was 0.07 mg/100 g DW and catechin 65.16 mg/100 g DW (Table 2). In the extracts from the microshoots grown in PlantForm bioreactors, the catechin content was 124.25 mg/100 g DW, protocatechuic acid was 0.07 mg/100 g DW, and catechin was 65.16 mg/100 g DW (Table 2).

### Total polyphenol content (TPC)

The highest TPC was found for agar microshoot and microshoots maintained in bioreactor, which were 1240.51 and 598.65 mg GAE/100 g DW, respectively. Lower TPC was found for biomass extracts from agitated microshoot and suspension cultures, which were 557.96 and 457.88 mg GAE/100 g DW, respectively. The lowest concentration of polyphenolic compounds was observed for callus culture (317.32 mg GAE/100 g DW). The results are compatible to the results obtained during the evaluation of the total antioxidant potential by DPPH and FRAP methods as well as for the chelating ability assay (Table 3).

**Table 3:** Results of total polyphenol content and antioxidant potential of the tested types of *S. henryi* in vitro cultures

In vitro culture	TPC (mg GAE/100 g DW ± SD)	Total antioxidant potential by DPPH (% of inhibition ± SD)	Total antioxidant potential by FRAP methods (uM/L TEAC ± SD)	Chelation ability (FIC) (%)
Callus	317.32 ± 21.01	52.49 ± 0.69	19.81 ± 0.76	44.80 ± 3.35
Suspension	457.88 ± 14.85	66.05 ± 0.49	34.69 ± 1.31	50.22 ± 1.00
Agar microshoot	1240.51 ± 18.28	90.98 ± 0.01	59.31 ± 4.34	88.89 ± 0.65
Agitated microshoot	557.96 ± 29.71	75.54 ± 2.25	42.79 ± 1.48	57.50 ± 2.64
Microshoots in bioreactor	598.65 ± 10.16	76.13 ± 2.54	53.29 ± 0.71	± 1.13
Standard compound	-	BHT 96.52 ± 0.72	-	EDTA 99.80 ± 0.10

### Antioxidant potential

The antioxidant potential measured by the DPPH assay showed the highest results for agar microshoot extracts, as well as microshoots maintained in PlantForm bioreactors which have shown 90.98% and 76.13% inhibition. respectively. A lower antioxidant potential was demonstrated for the extracts from agitated microshoot and suspension which were 76% and 66% respectively. The lowest total antioxidant potential was determined for agar callus which was 52%. The synthetic antioxidant BHT was used as the standard at a concentration of 2 mg/mL. Its activity reached 97%, which is a value comparable to the ability of extracts from microshoot to reduce free radicals. This indicates a high activity level of extracts obtained from this material. Importantly, the study shows that the extracts exhibit high antioxidant power (Fig. S2-S5).

The FRAP assay results were compatible with the DPPH. The ability to reduce reactive oxygen species showed that the most potent extract was obtained from agar microshoots (59.31 μM/L TEAC), similarly to chelating ability which for this extract reached the value of 89%. The less potent extract was from callus (19.81 μM/L TEAC). The extracts obtained from suspension, agitated microshoots, and bioreactors exhibited moderate antioxidant and chelating activity. The examined extracts exhibited fairly high antioxidant potential, effectively reducing free radicals and also reducing transition metals.

## Discussion

Plant biotechnology is a field of science that is developing more and more dynamically and is becoming an alternative to obtaining plant metabolites compared to species growing in vivo. In vitro plant cultures are also characterized by the possibility of developing protocols that will increase the production of selected secondary metabolites by optimizing the cultivation conditions (Pietrosiuk and Furmanowa 2006). The research presented in the article is the first report on the cultivation of suspension, shaken, and microshoot cultures of *S. henryi* grown in PlantForm bioreactors. In addition, for the first time, the analysis of polyphenols in extracts from various types of in vitro cultures (agar microshoots and callus, agitated, suspension, microshoots in PlantForm bioreactors) of *S. henryi* was carried out. The research is innovative on a global scale and expands knowledge about the chemical composition and biological activity of extracts from the species *S. henryi*, which may become an alternative to another pharmacological species of the *Schisandra* genus—*S. chinensis*. Qualitative UHPLC-DAD-ESI-MS^3^ analysis showed the presence of compounds from the group of polyphenols–phenolic acids and mainly procyanidins counting catechin as their precursor. These compounds have a number of therapeutic activities that are used in the pharmaceutical industry. *S. henryi* has not been previously studied for polyphenolic compounds, nor its derived in vitro cultures. The qualitative profiles of individual types of cultures differed in the number of estimated compounds. In undifferentiated *S. henryi*–callus agar and suspension cultures, they amounted to 18 and eight compounds from the polyphenol group, respectively. In microshoot cultures: agar, microshoot, and in PlantForm bioreactors, 14, 20, and 12 compounds were quantified, respectively (Table 1, Supplemental Fig. S1). This study confirmed that agar microshoot culture extracts are the richest source of polyphenols in quantitative terms, comparing the results to other culture types tested (Table 1). In extracts from microshoot agar cultures, quantitative analysis of four compounds was performed and confirmed by qualitative analyses: neochlorogenic acid, caffeic acid, protocatechuic acid, and catechin 16.54 mg/100 g DW and 112.56 mg/100 g DW (Table 2). Neochlorogenic acid (22.92 mg/100 g DW), protocatechuic acid (18.11 mg/100 g DW), and catechin (65.79 mg/100 g DW) were quantitatively confirmed in agitated cultures (Table 2). In extracts from cultures cultivated in PlantForm bioreactors, a quantitative analysis of one compound, catechin, was performed, which amounted to 124.25 mg/100 g DW. In extracts from undifferentiated cultures, quantitative analysis was performed for two compounds confirmed by qualitative analyses: protocatechuic acid and catechin (Table 2). In callus and suspension cultures, quantitative results for protocatechuic acid and catechin were 7.51 mg/100 g DW and 22.51 mg/100 g DW, respectively, 0.07 mg/100 g DW and 65.16 mg/100 g DW (Table 2). Judging by the results obtained, agar microshoot cultures may be a source of obtaining selected compounds from polyphenols, which may contribute to the use of *S. henryi* cultures on a global and industrial scale. In almost all extracts from various types of cultures (except microshoots in the PlantForm bioreactor) the presence of protocatechuic acid, a phenolic acid with a wide therapeutic effect, was found, including antioxidant, anti-inflammatory, and cardioprotective activity which was confirmed in a study. This creates prospects for the use of extracts from in vitro cultures of *S. henryi* in obtaining this compound on a large scale. An interesting group of compounds that have been qualitatively determined in extracts are also procyanidins—polyphenols whose basic monomeric structures are catechin and epicatechin. Procyanidins have anticancer, anti-inflammatory, and antioxidant properties. The largest amounts of various types of procyanidins were found in extracts from *S. henryi* suspension cultures, which creates a number of possibilities for using the extracts to obtain these valuable compounds. Additionally, this is the first report that confirms the presence of procyanidins in extracts from in vitro cultures of *S. henryi*. An important aspect is also a number of studies on procyanidins with a neuroprotective effect, which opens new opportunities for innovative research and the use of culture extracts as plant medicines in neurodegenerative diseases.

For the first time on a global scale, antioxidant power tests were performed using the FRAP and DDPH methods and the chelating capacity (FIC) was determined. It was found that the extract from microshoot agar cultures also had the highest antioxidant power (DDPH, FRAP, and FIC), which is consistent with the qualitative and quantitative analyses of the tested extracts. Antioxidant potential by DPPH, FRAP, and chelating ability for extracts from microshoot agar cultures amounted to 90% of inhibition, 59.31 nM/L TEAC, and 89%, respectively. The antioxidant potential of extracts from agitated cultures using the DPPH and FRAP methods and the chelating capacity were 76% of inhibition, 42.79 nM/L TEAC, and 58%, respectively, and were lower by 1.19, 1.38, and 1.54 times respectively, compared to the results from microshoot agar extracts. Antioxidant power determined by DPPH, FRAP, and FIC methods for extracts from cultures grown in PlantForm bioreactors was 76% of inhibition, 53.29 nM/L, and 81%, respectively, and was lower by 1.19, 1.11, and 1.10 times respectively compared to the results from microshoot agar extracts. In extracts from undifferentiated cultures, the antioxidant power and chelating capacity were relatively lower compared to the results from extracts from microshoot cultures, which also correlates with the results from qualitative and quantitative analyses. The results of DPPH, FRAP, and FIC assays for callus agar extracts are 52% of inhibition, 19.81 nM/L TEAC, and 45%, respectively, and are lower by 1.73, 2.99, and 1.98 times, respectively, compared to the results from microshoot agar extracts. The results of antioxidant potential for extracts from suspension cultures were equal to 66% of inhibition, 34.69 nm/L, and 50%, respectively, and were 1.38, 1.71, and 1.77 times lower, respectively, compared to the results obtained in extracts from agar microshoot cultures.

As for the antioxidant potential, also in the case of TPC, the highest results were indicated for microshoot types of cultures, which clearly correlated with the results of qualitative and quantitative analysis of the tested polyphenolic compounds. The highest TPC was found for extracts from microshoot agar cultures—1240.51 mg GAE/100 g DW. In extracts from agitated cultures and those grown in PlantForm bioreactors, the TPC was 557.96 and 598.65 mg GAE/100 g DW, and the contents were 2.22 and 2.07 times lower, compared to the results from agar microshoot cultures extracts. The results of TPC from extracts from undifferentiated cultures were significantly lower; the TPC in extracts from callus agar and suspension cultures was 317.32 mg GAE/100 g DW and 457.88 mg GAE/100 g DW, respectively. Their contents were 3.90 and 2.71 times lower than the results from extracts from microshoot agar cultures (Fig. S3).

For all measurements of total antioxidant potential and determination of total polyphenol content, the results obtained indicate that agar microshoot culture types have the greatest ability to reduce free radicals. This is an activity that effectively fights free radicals and neutralizes their destructive impact. Studies have shown the correlation between the content of polyphenolic compounds in extracts and their antioxidant potential and TPC (Fig. S3).

**Fig. 2:**
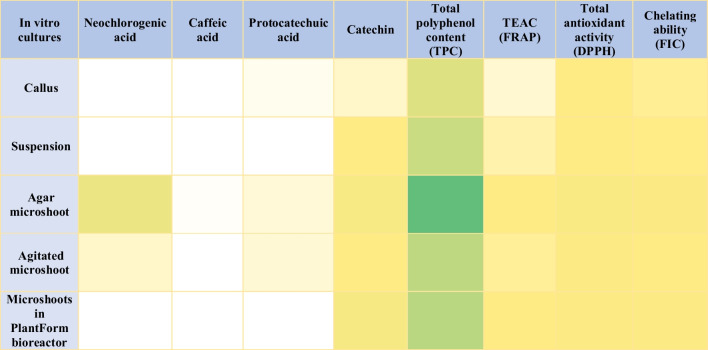
Heatmap comparing the quantitative analysis and antioxidant potential results of *S. henryi* in vitro cultures extracts. The heatmap illustrates the varying levels of different compounds across different sample types in a biological study. The color gradient reflects the concentration or presence of specific compounds (neochlorogenic acid, caffeic acid, protocatechuic acid, catechin), total polyphenol content (TCP), TEAC (by FRAP method), total antioxidant potential (by DPPH method), and chelating ability (FIC), within each sample type

Agar callus extracts show a significant concentration of protocatechuic acid and catechin compared to other compounds, with moderate levels of TPC and antioxidant potential. Biomass suspension cultures show a notably high concentration of TPC and significant levels of catechin and chelating ability. Agar microshoot extracts demonstrate high concentrations of neochlorogenic acid, total polyphenol content, and TEAC. Agitated microshoots present moderate levels of all compounds, especially in protocatechuic acid, total polyphenol content, and antioxidant potential. Microshoots in bioreactors have considerable levels of catechin, total polyphenol content, TEAC, and chelating ability compared to other compounds. The heatmap in Fig. 2 enables a visual comparison of the sample types in terms of their composition of these compounds, providing a quick and clear understanding of the variations in compound concentrations across the different biological samples studied.

The results of this work can be compared with previous studies carried out on the species *S. henryi* regarding the analysis of the chemical profile of only extracts from microshoot and callus cultures grown on agar (Jafernik et al. 2020). Based on this previous study, several variations of the culture medium were used in order to select one that would increase the production of secondary metabolites as well as the biomass growth of in vitro cultures. The study determined both the phenolic profile and the lignan content using the HPLC–DAD method. The seven out of 24 tested phenolic acids were quantified in extracts from microshoot cultures, the amounts of which ranged from 0.17 to 370.81 mg/100 g DW, and in six of 14 tested flavonoids, the amounts of which ranged from 2.35 to 138.56 mg/100 g DW. Comparing the same variant of the culture medium and the duration of culture used in the former and present study, the estimated contents of neochlorogenic acid and caffeic acid before were lower by 1.62 and 1.61 times, respectively. In the tested extracts from callus cultures, the same phenolic acids were identified as in microshoot cultures, the amounts of which ranged from 0.07 to 187.91 mg/100 g DW. In previous studies, the presence of catechin and protocatechuic acid was not detected.

The former study also involved the HPLC–DAD assays of phenolic profiles in extracts from the leaves of the parent plant collected from two growing seasons (May and September). The following phenolic acids: gallic, neochlorogenic, caftaric, protocatechuic, and caffeic, were quantitatively confirmed, the contents of which ranged from 3.05 to 46.64 mg/100 g DW from leaf extracts collected in May and from 5.00 to 64.16 mg/100 g DW collected in September. For the flavonoid profile, hyperoside, rutoside, trifolin, quercitrin, quercetin, and kaempferol were identified. The quantitative content from extracts collected in May ranged from 25.15 to 134.58 mg/100 g DW, and from September, ranged from 3.62 to 128.87 mg/100 g DW. The amount of neochlorogenic acid was 46.64 mg/100 g DW (May) and 37.87 mg/100 g DW (September); protocatechuic acid was 10.23 mg/100 g DW (May) and 10.79 mg/100 g DW (September) and caffeic acid was 3.05 mg/100 g DW (May) and 9.38 mg/100 g DW (September). Comparing the achieved results of neochlorogenic acid with extracts from the leaves of the parent plant, it turns out that the content of neochlorogenic acid was 4.50 (May) and 5.55 (September) lower compared to the results from extracts from agar microshoot cultures and 2.04 (May) and 1.66 times higher compared to the results from extracts from agitated cultures. As for the quantitative content of protocatechuic acid in extracts from the leaves of the parent plant (May and September), the content was lower by 1.61 (May) and 1.53 (September) times, than in extracts from agar microshoot culture and 1.77 (May) and 1.68 (September) times lower than in extracts from agitated culture. The content of protocatechuic acid in extracts from the parent plant was 1.36 (May) and 1.43 (September) times higher than in extracts from agar callus culture and 146.14 (May) and 154.14 (September) than in suspension culture.

Szopa et al. (Szopa et al. 2017) conducted research on the polyphenol profiles of extracts from microshoot agar and agitated cultures of pharmacopeial species from the Schisandraceae family, *S. chinensis*, and its cultivar variety *S. chinensis* cv. Sadova. The polyphenol profile for extracts from both types of cultures were the same. The presence of seven phenolic acids (chlorogenic, gallic, p-hydroxybenzoic, protocatechuic, salicylic, syringic, vanillic acids) and three flavonoids (kaempferol, quercitrin, rutoside) was confirmed. No neochlorogenic or caffeic acid was detected in microshoot agar and agitated cultures. Protocatechuic acid was identified in extracts from microshoot agar and agitated cultures; the amount measured was 35.69 mg/100 g DW and 14.14 mg/100 g DW, respectively. The amounts shown in extracts from microshoot agar cultures of *S. chinensis* were higher by 2.15, 1.97, 4.75, and 509.85 times than the results obtained from microshoot agar, agitated, callus agar, and suspension cultures of *S. henryi*. The presented amounts in extracts from agitated cultures of *S. chinensis* were respectively lower by 1.17 and 1.29 than the results obtained in extracts from microshoot agar and agitated cultures and lower by 1.88 and 2.02 respectively than the results obtained from extracts from callus agar and suspension cultures of *S. henryi*. No catechin was found in extracts from *S. chinensis* agitated microshoot agar cultures.

Szopa et al. (Szopa et al. 2019b) in extracts from *S. chinensis* cv. Sadova microshoot culture confirmed the presence of eight phenolic acids (chlorogenic, cryptochlorogenic, gallic, neochlorogenic, protocatechuic, salicylic, syringic, vanillic acids) and two flavonoids (kaempferol, quercitrin). The polyphenol profiles for the three types of cultures tested: microshoot agar, agitated agar, and cultured in PlantForm bioreactors, were the same. Neochlorogenic acid was quantitatively determined in the following amounts: 116.65 mg/100 g DW, 91.33 mg/100 g DW, and 100.26 mg/100 g DW for extracts from microshoot agar, agitated cultures, and those grown in PlantForm bioreactors. The obtained values were 1.80, 2.30, and 2.09 times lower than those obtained from extracts from agitated microshoot cultures of *S. henryi* and were found to be higher by 5.11, 4, and 4.39 times than the results obtained in extracts from agitated cultures of *S. henryi*. As for the quantification of protocatechuic acid, the values were 53.81, 28.39, and 6.40 mg/100 g DW for extracts from for the following cultures: microshoot agar, agitated, and PlantForm bioreactors of *S. chinensis* cv. Sadova. Protocatechuic acid values obtained from microshoot agar of S. chinensis cv. Sadova were respectively higher by 3.25, 18.71, 7.16, and 768.71 times the results obtained from extracts from of microshoot agar, agitated, callus agar, and suspension cultures of *S. henryi*. The results obtained for protocatechuic acid from agitated cultures of *S. chinensis* cv. Sadova were higher by 1.72, 1.56, 3.51, and 405.57 times than the results obtained from microshoot agar, agitated microshoot, callus, and suspension cultures of *S. henryi*. The obtained values for protocatechuic acid obtained from extracts from of S. chinensis cultures grown in PlantForm bioreactors were lower by 2.58, 2.82, and 1.17 times, compared to the results obtained from extracts from of agar microshoot, agitated, and callus cultures. The result was 91.34 times higher for S. chinensis compared to suspension cultures of *S. henryi*.

The antioxidant potential and determination of the TPC of extracts from various types of cultures with *S. henryi* were examined for the first time, which makes comparative analysis very difficult.

A large number of publications are based on research on the polyphenol profile of extracts from different types of in vitro cultures. The authors focus on the qualitative and quantitative determination of polyphenols, also examining antioxidant potential (Aksenova et al. 2023; Zheleznichenko et al. 2023; Kwiecień et al. 2023).

The in vitro cultures—agar microshoot and PlantForm bioreactors—of three varieties of the species *Hypericum perforatum* (Elixir, Helos, Topas) were tested for polyphenol profiles. Extracts from *H. perforatum* microshoot cultures contained seven phenolic acids, including neochologenic acid, three flavonoids, and two flavonols, including catechin. The highest total amounts of phenolic acids and flavonoid were found for the Helos variety (505 and 2386 mg/100 g DW, respectively). The content of flavonols was the highest in the Elixir variety agar microshoot cultures (712 mg/100 g DW). Extracts from cultures grown in PlantForm bioreactors contained six phenolic acids, including neochlorgenic acid, six flavonoids, and two flavonols, including catechin. The highest maximum total content of phenolic acids, flavonoids, and flavonols was found for the Topas variety (1101 mg/100 g DW), followed by the Helos variety (375 mg/100 g DM) and the Elixir variety (336 mg/100 g DW). The TPC was also tested using the F–C method. The TPC in *H. perforatum* biomass extracts ranged from 69 to 76 mg GAE/g. The Helos variety contained the highest polyphenol content, 75.99 mg GAE/g of extract (Kwiecień et al. 2023). Comparing the results obtained from extracts from different types of *S. henryi* to the results from extracts from different varieties of *H. perforatum*, it contained more polyphenol compounds from the group of phenolic acids, flavonoids, and flavonols. Total content of polyphenol extracts from microshoot cultures of *H. perforatum* was similar to the results obtained from microshoot cultures of *S. henryi*. This may be due to the presence of other compounds with high antioxidant power present in the in vitro cultures of *S. henryi* like dibenzocyclooctadiene lignans (Jafernik et al. 2020).

Zheleznichenko et al. tested the content of polyphenolic compounds in the *Sorbaria pallasii* in microshoot culture. The maximum contents of phenolcarboxylic acids, flavonols, and tannins in the extracts were measured and found to be 30.8 mg/g DW, 4.3 mg/g DW, and 74.9 mg/g DW, respectively. The highest content was found for tannins. Furthermore, the presence of three compounds from the catechin group was found, including catechin, the content of which was 3.03 mg/g ADM in extracts from microshoot cultures. The anti-free radical potential was tested using the DPPH reagent, which was IC_50_ = 589.66 µg/mL. The TPC was also determined using the F–C method, which amounted to 30.2 mg/g ADM (Zheleznichenko et al. 2023).

Aksenova et al. focused on the analysis of the phytochemical profile of in vitro callus cultures of *Camellia sinensis*. Different precursors were added to increase the accumulation of secondary metabolites. The research revealed the presence of polyphenolic compounds from the group of phenylpropanoids, flavans, and proanthocyanidins and confirmed that the addition of precursors such as phenylalanine significantly increased the accumulation of the compounds (Aksenova et al. 2023).

As a result of the research based on in vitro cultures of various species, this study has shown that extracts from *S. henryi* microshoot cultures are the valuable source of polyphenolic compounds, which are shown to have a very strong antioxidant potential.

This reveals that maintaining and utilizing *S. henryi* in vitro cultures may create an opportunity for a new direction of research in the field of biotechnology to create new and innovative plant material with a high medicinal utility.

## Supplementary Information

Below is the link to the electronic supplementary material.

Supplementary file1 (PDF 654 KB)

## Data Availability

Raw data were generated at Department of Pharmaceutical Botany, Faculty of Pharmacy, Jagiellonian University, Medical College; Department of Organic Chemistry and Technology, Faculty of Chemical Engineering and Technology, Cracow University of Technology; and Department of Pharmacognosy and Molecular Basis and Phytotherapy, Medical University of Warsaw (Poland). The data that support the findings of this study are available on request from the first (K.J.) and corresponding (A.S.) authors. The authors confirm that the data supporting the findings of this study are available within the article.
