# Supplementary material for: Phenolic compound profiling and antioxidant potential of different types of Schisandra henryi in vitro cultures
Source: Appl Microbiol Biotechnol. 2024 May 7;108:322. doi: 10.1007/s00253-024-13159-6 (PMC11076313; doi:10.1007/s00253-024-13159-6)
Supplement: Supplementary file 1 [file 253_2024_13159_MOESM1_ESM.pdf]

Applied Microbiology and Biotechnology

SUPPLEMENTARY DATA

Phenolic compounds profiling and antioxidant potential of different types of *Schisandra henryi* in vitro cultures

Karolina Jafernik[1], Paweł Kubica[1], Marta Sharafan[2], Aleksandra Kruk[3], Magdalena Anna Malinowska[2], Sebastian Granica[3], Agnieszka Szopa[1*]

[1]Chair and Department of Pharmaceutical Botany, Jagiellonian University, Medical College, Medyczna 9 Str, 30-688 Cracow, Poland
[2]Department of Organic Chemistry and Technology, Faculty of Chemical Engineering and Technology, Cracow University of Technology, Warszawska 24 Str, 31-155 Cracow, Poland
[3]Department of Pharmacognosy and Molecular Basis and Phytotherapy, Medical University of Warsaw, Banacha 1 Str, 02-097 Warsaw, Poland
*correspondence: a.szopa@uj.edu.pl, +48-126-205-430 (A.S.)

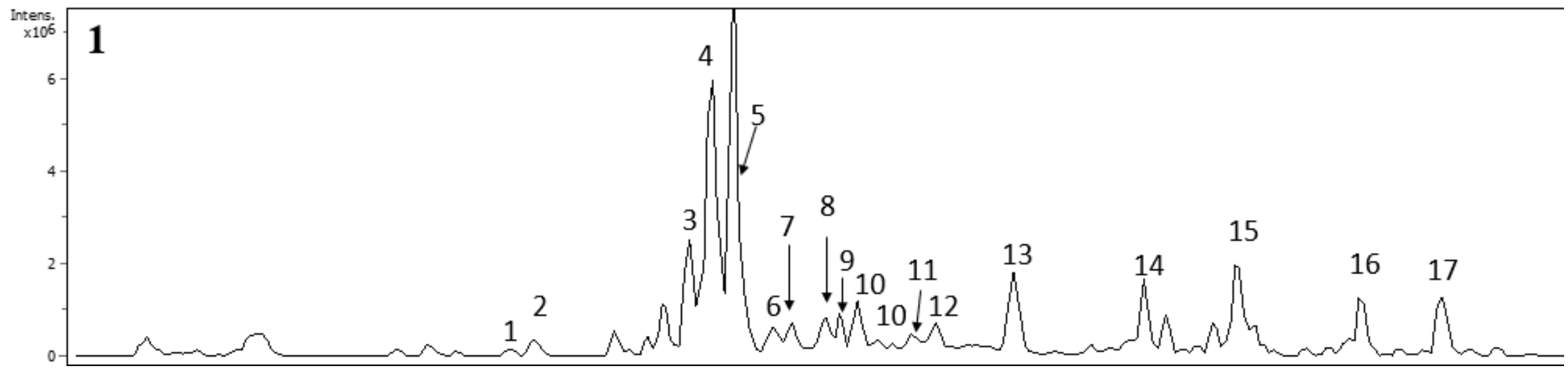

1. protocatechuic acid *O*-hexoside 2. procyanidin trimer type C isomer 3. coumaroylquinic acid isomer; procyanidin dimer type B isomer; procyanidin dimer type B isomer 5. Catechin 6. procyanidin trimer type C isomer; procyanidin dimer type B isomer 7. procyanidin tetramer isomer 8. procyanidin tetramer isomer 9. coumaroylquinic acid isomer 10. procyanidin dimer type B isomer, procyanidin trimer type C isomer 11. procyanidin tetramer isomer 12. procyanidin dimer type B isomer; procyanidin trimer type C isomer 13 – 18. unknown

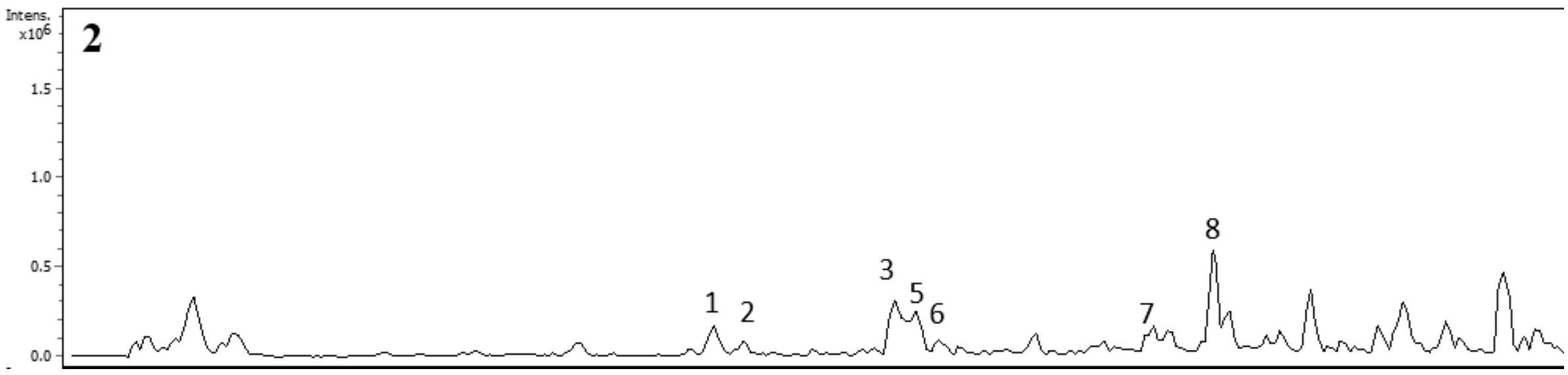

1. procyanidin dimer type B isomer 2. catechin 3-8. unknown

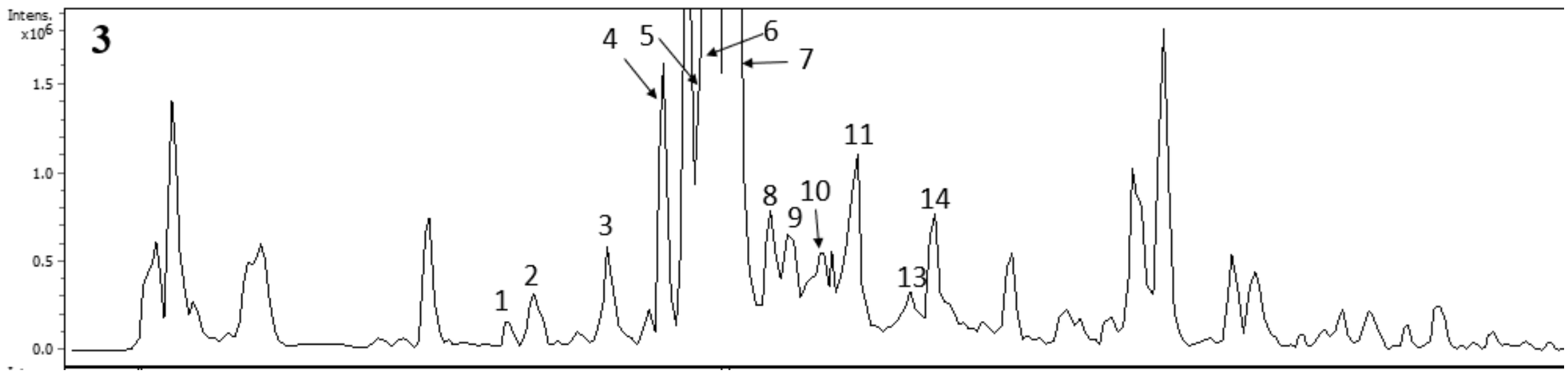

1. protocatechuic acid *O*-hexoside 2. procyanidin trimer type C isomer 3. neochlorogenic acid 4. procyanidin trimer type C isomer 5. coumaroylquinic acid; procyanidin dimer type B isomer 6. procyanidin dimer type B isomer 7. catechin 8. caffeic acid derivative; procyanidin trimer type C isomer 9. procyanidin tetramer isomer 10. procyanidin tetramer isomer 12. procyanidin dimer type B isomer; procyanidin trimer type C isomer 13. procyanidin tetramer isomer 14. procyanidin dimer type B isomer; procyanidin dimer type B isomer

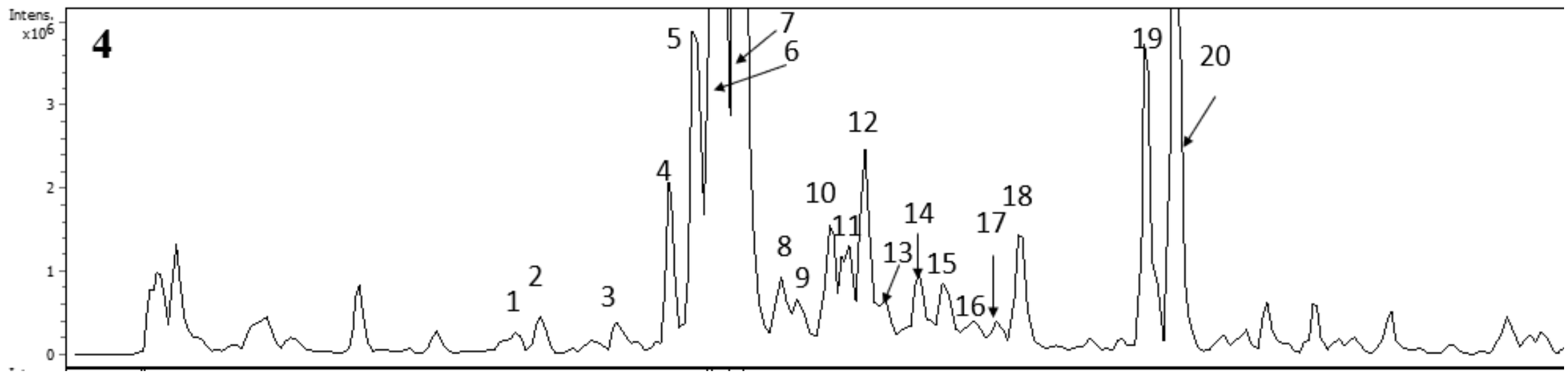

1. protocatechuic acid *O*-hexoside **2.** procyanidin trimer type C isomer **3.** neochlorogenic acid; procyanidin dimer type A isomer **4.** unknown; procyanidin trimer type C isomer **5**. coumaroylquinic acid isomer; procyanidin dimer type B isomer **6.** procyanidin dimer type B isomer; procyanidin tetramer isomer **7.** catechin **8.** procyanidin trimer type C isomer **9.** procyanidin tetramer isomer **10.** procyanidin tetramer isomer **11.** procyanidin dimer type B isomer; coumaroylquinic acid isomer **12**. procyanidin dimer type B isomer; procyanidin trimer type C isomer **13.** procyanidin pentamer isomer **14.** procyanidin tetramer isomer **15.** procyanidin dimer type B isomer; procyanidin trimer type C isomer **16.** procyanidin tetramer isomer **17.** procyanidin tetramer isomer **18.** unknown; procyanidin pentamer isomer **19- 20**. unknown

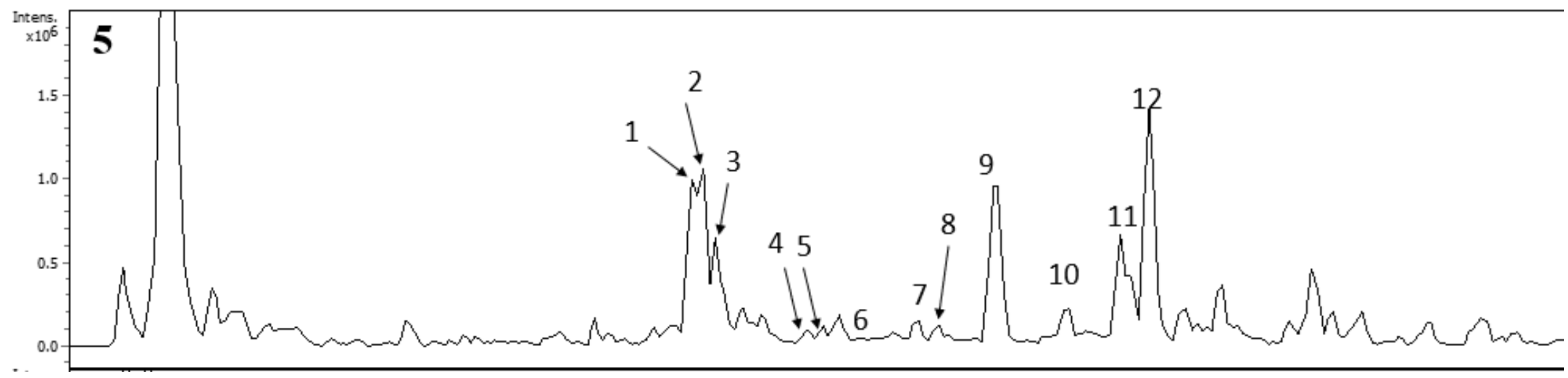

1. procyanidin dimer type B isomer 2. unknown; procyanidin dimer type B isomer 3. catechin 4. procyanidin tetramer isomer 5. coumaroylquinic acid 6.procyanidin dimer type B isomer; procyanidin trimer type C isomer 7. procyanidin dimer type B isomer 8. procyanidin tetramer isomer 9-12. unknown

Figure S1. UHPLC-DAD-ESI-MS^3 chromatograms of extracts from biomass of various types of *S. henryi in vitro* cultures: 1. agar callus cultures, 2. suspension cultures, 3. microshoot agar cultures, 4. agitated microshoot cultures, 5. microshoots in bioreactors.

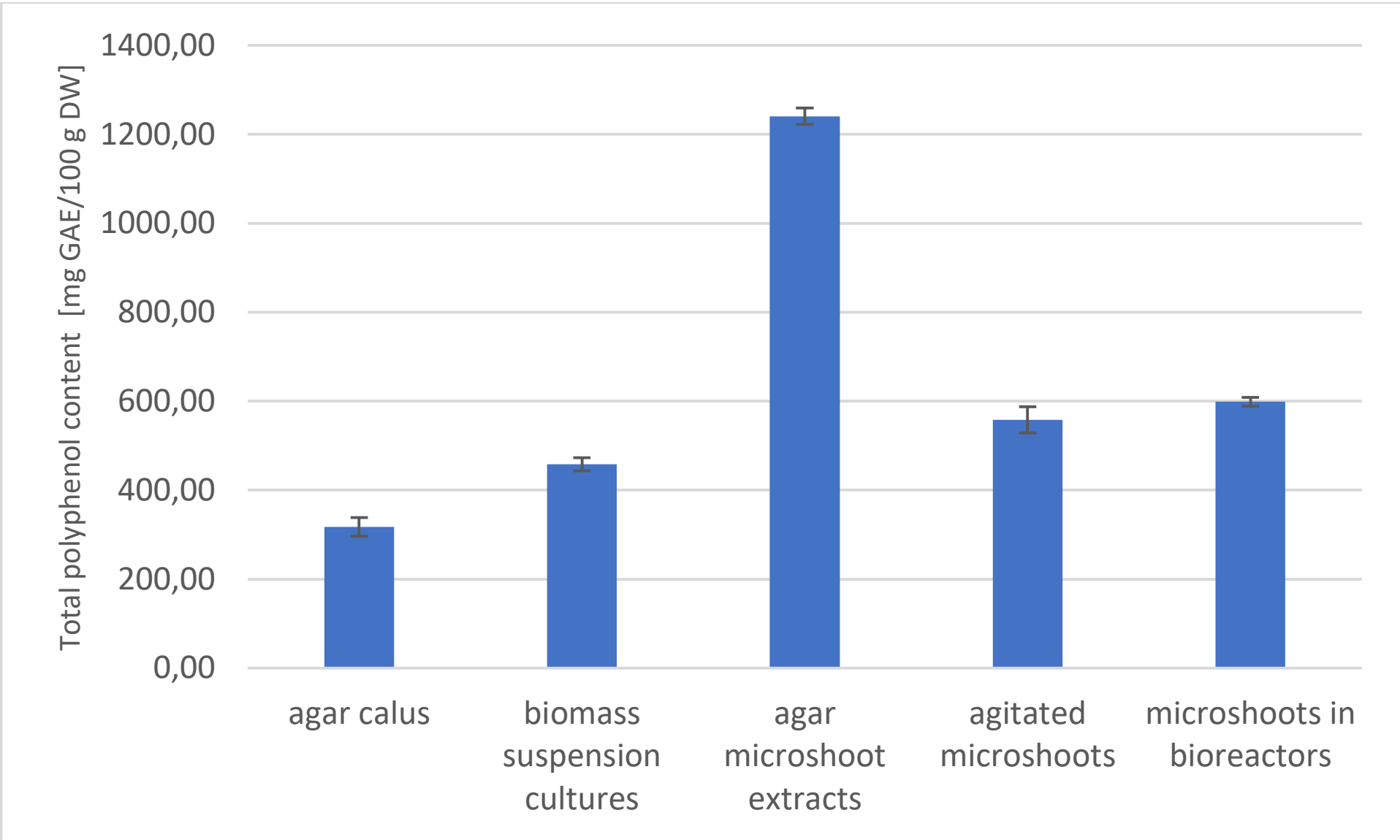

Figure S2. The total polyphenol content (TPC) of the extracts tested.

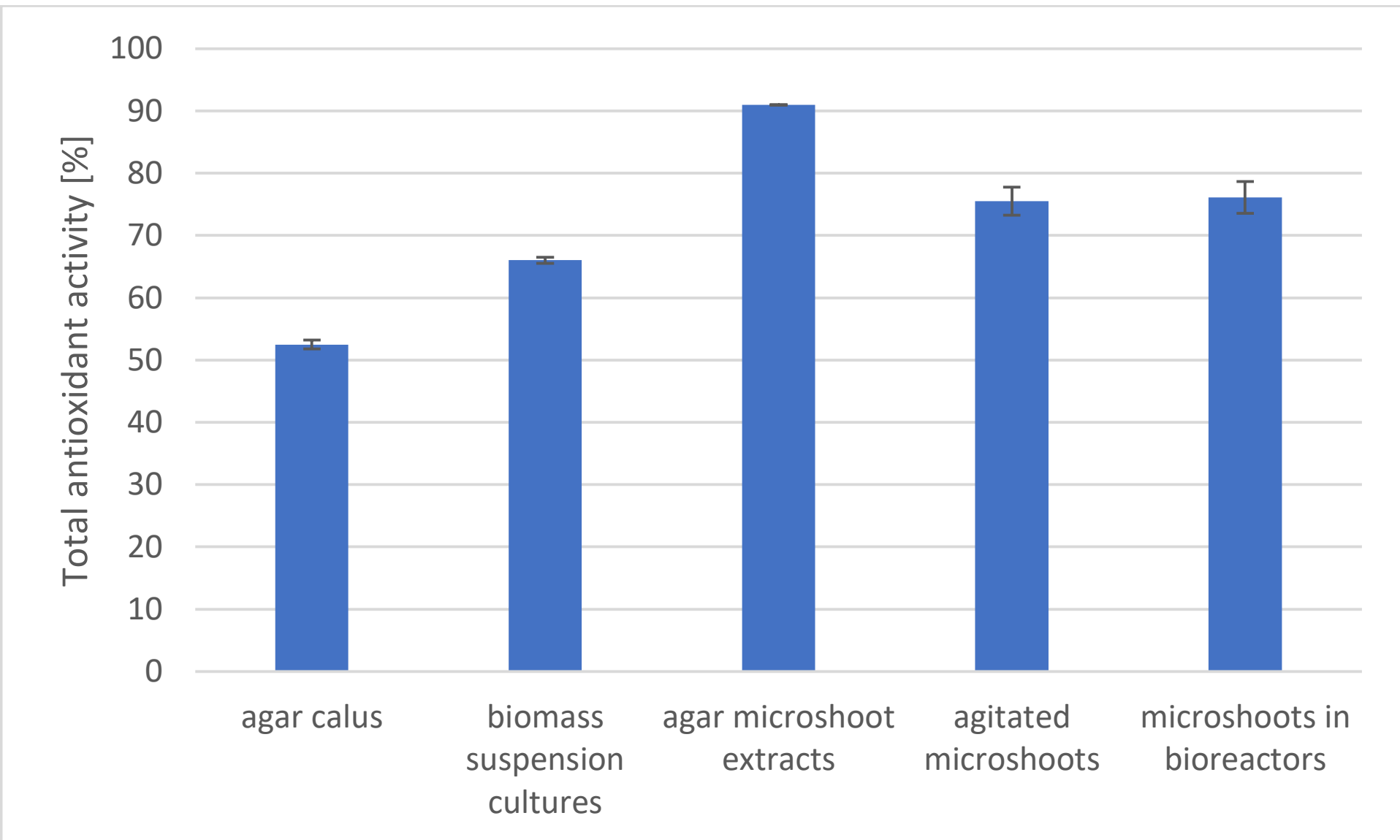

Figure S3. The total antioxidant activity expressed as % of oxidation inhibition (evaluated by DPPH method)

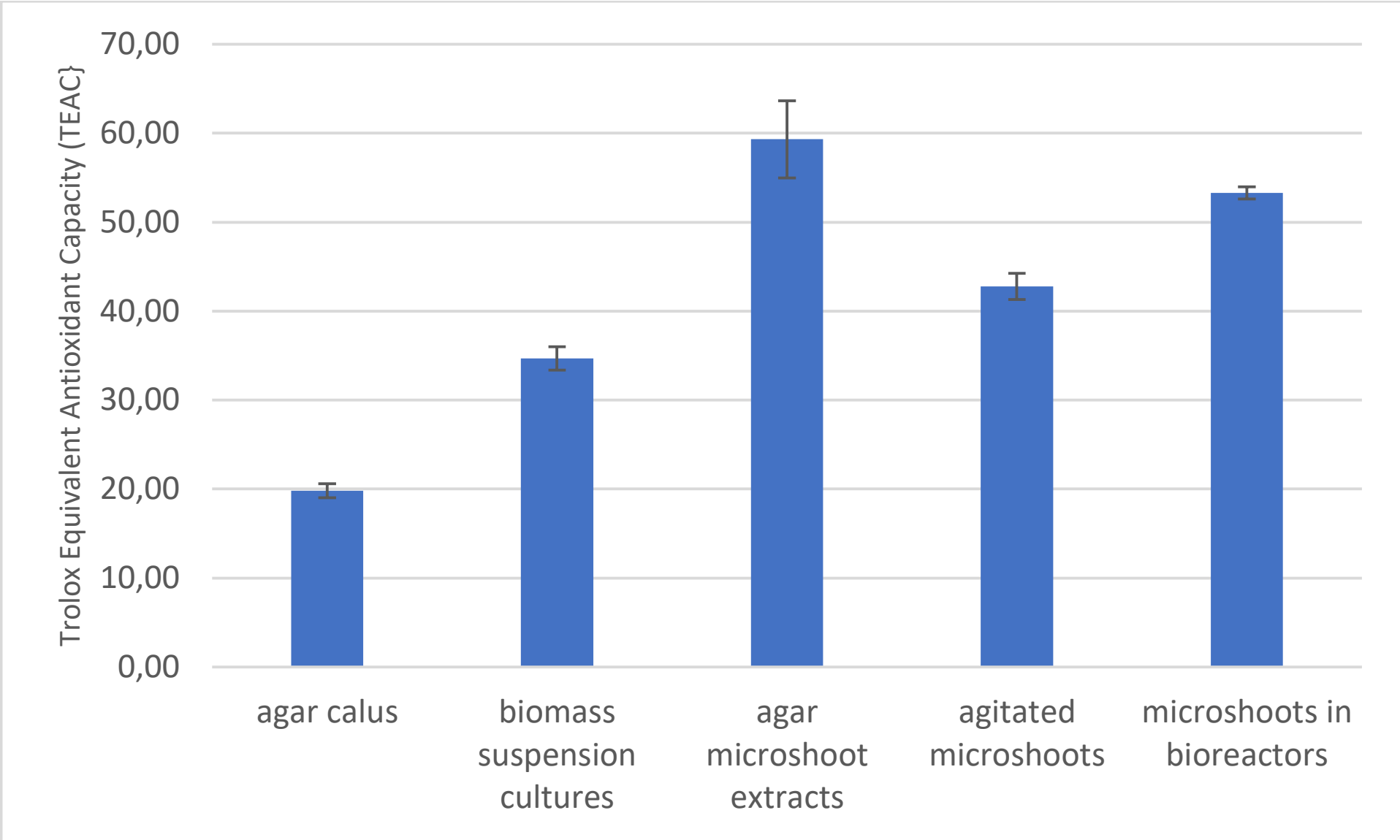

Figure S4. The total antioxidant activity presented as TEAC (determined by FRAP).

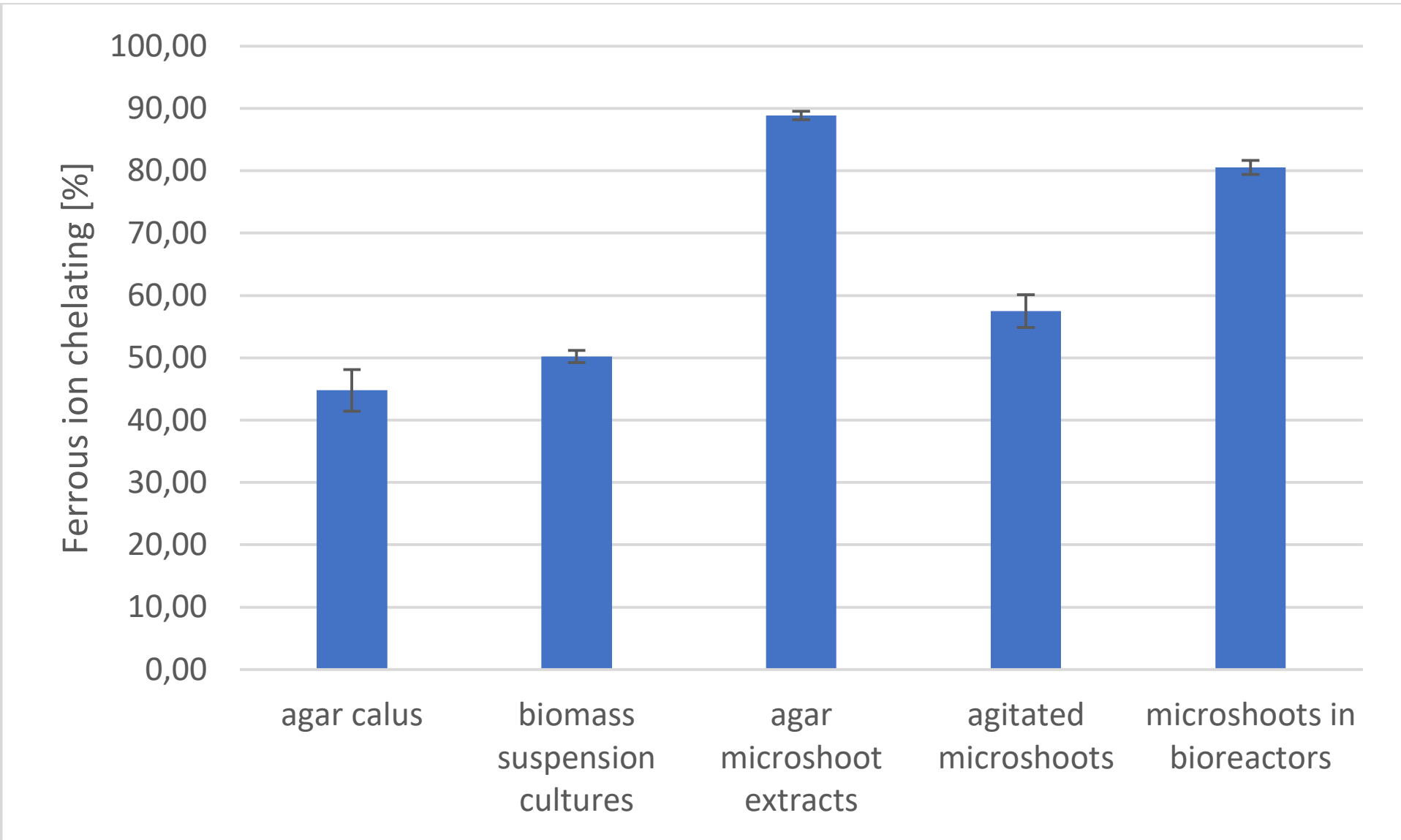

Figure S5. The ferrous ion chelating activity (FIC) results of the extracts tested.
